# Enhanced crystallinity and electrical properties through homoepitaxial growth on reduced graphene oxide templates

**DOI:** 10.1038/s41598-026-54930-1

**Published:** 2026-05-30

**Authors:** S. Kanda, T. Yamashita, S. Kurosu, F. Sakamoto, T. Hanajiri, Y. Nishina, R. Negishi

**Affiliations:** 1 Graduate School of Science and Engineering, Toyo Univ., 2100, Kujirai, Kawagoe-city, Saitama 350-8585 Japan; 2 Bio-nanoelectronics Research Centre, 2100, Kujirai, Kawagoe-city, Saitama 350-8585 Japan; 3 Research Institute for Interdisciplinary Science, Okayama Univ, 3-1-1 Tsushimanaka, Kita-ku, Okayama-city, Okayama 700-8530 Japan

**Keywords:** Energy science and technology, Materials science, Nanoscience and technology, Physics

## Abstract

Graphene oxide (GO), which can be synthesized inexpensively and in large quantities, is regarded as a promising starting material for electronic device applications due to its ability to recover electrical conductivity through reduction. However, oxygen-containing functional groups and structural defects introduced during the oxidation and reduction process significantly impair the electrical performance of reduced graphene oxide (rGO), posing a major challenge for practical implementation. In this study, we demonstrate that high-temperature thermal reduction in the presence of a carbonaceous gas not only facilitates the repair of vacancies in rGO thin films but also induces the homoepitaxial growth of two-dimensional graphene islands, guided by the underlying rGO template. By precisely controlling the growth driving force of the carbonaceous gas, epitaxial graphene islands were successfully formed, resulting in a significant improvement in electrical performance, with Hall mobilities reaching up to 365 cm^2^/V·﻿s. These results suggest that the homoepitaxial growth of graphene islands plays a crucial role in enhancing both the crystallinity and electrical properties of rGO thin films.

**Supplementary Information:**

The online version contains supplementary material available at 10.1038/s41598-026-54930-1.

## Introduction

Graphene oxide (GO) is a scalable two-dimensional material that can be synthesized at low cost and with high yield^1,2^. Owing to its potential applications in a wide range of electronic devices such as sensors, transparent conductive films and supercapacitors, GO has attracted significant attention in recent years for both its fundamental properties and practical utility^3–7^. GO is typically produced by oxidizing graphite, a process that increases the interlayer spacing and weakens the van der Waals forces between the layers^2^. The most common method to chemically exfoliate into single-layer GO is the modified Hummers method^2^. The resulting GO can be deposited onto various device substrates to form large-area GO thin films. However, GO exhibits insulating behavior due to the presence of numerous oxygen-containing functional groups and structural defects. Therefore, a reduction process is essential to restore its electrical conductivity for use in electronic devices.

Several reduction methods have been explored for GO, including chemical reduction using agents^8–11^ such as hydrazine, photoreduction under light irradiation^12–14^, and thermal reduction conducted in inert atmospheres^15,16^ such as argon. Among these approaches, thermal reduction has proven particularly effective in removing oxygen-containing functional groups, such as hydroxyl, carboxyl, and carbonyl moieties, thereby yielding reduced graphene oxide (rGO) with improved electrical conductivity^13,16–18^.

Nevertheless, previous studies have shown that structural defects introduced during the oxidation process, such as vacancies, 5- and 7-membered rings, and dislocations, are not repaired by reduction treatments and remain embedded within the graphene lattice^19,20^. These structural defects disrupt the intrinsic hexagonal lattice, thereby degrading the electrical transport properties of the graphene.

Plasma and high-temperature thermal reduction of GO have been extensively investigated under carbonaceous atmospheres such as methane^21–23^ and ethanol^24–27^. Under these conditions, carbonaceous gases decompose into reactive carbon precursors through plasma irradiation or high-temperature thermal reduction, which react with dangling bonds at vacancies and thereby promote partial repair of structural defects^21–27^. Alcohol-based carbon sources such as ethanol differ from hydrocarbon gases in that they generate hydroxyl radicals during thermal decomposition. These hydroxyl radicals are known to suppress the formation of amorphous carbon in carbon aggregates by an etching effect^28^. Indeed, high-temperature thermal reduction using ethanol has been reported to dramatically enhance the crystallinity of rGO with increasing reduction temperature^24–26^, suggesting its effectiveness not only in removing oxygen-containing functional groups but also in repairing vacancies.

However, under excessive conditions, ethanol decomposition proceeds beyond the optimal range, leading not only to carbon-precursor-mediated repair of vacancies but also to the deposition of amorphous carbon on the rGO surface^27,29^. Thus, thermal reduction in an ethanol atmosphere intrinsically involves a trade-off by simultaneously enabling repair of vacancies that improves crystallinity while inducing amorphous carbon deposition as a side reaction, thereby making process control critically important. Nevertheless, the influence of surface morphological evolution on the crystallization behavior of rGO during high-temperature thermal reduction in an ethanol atmosphere remains insufficiently understood.

In this study, we systematically varied the reduction temperature and ethanol supply to investigate the relationship between crystallinity and surface morphology in rGO thin films. Our results reveal that the formation of highly crystalline rGO film is primarily driven by a homoepitaxial growth mechanism, which plays a central role in enhancing the film’s crystallinity.

## Results

### High crystallinity of rGO by thermal reduction in an ethanol atmosphere

**Fig. 1:**
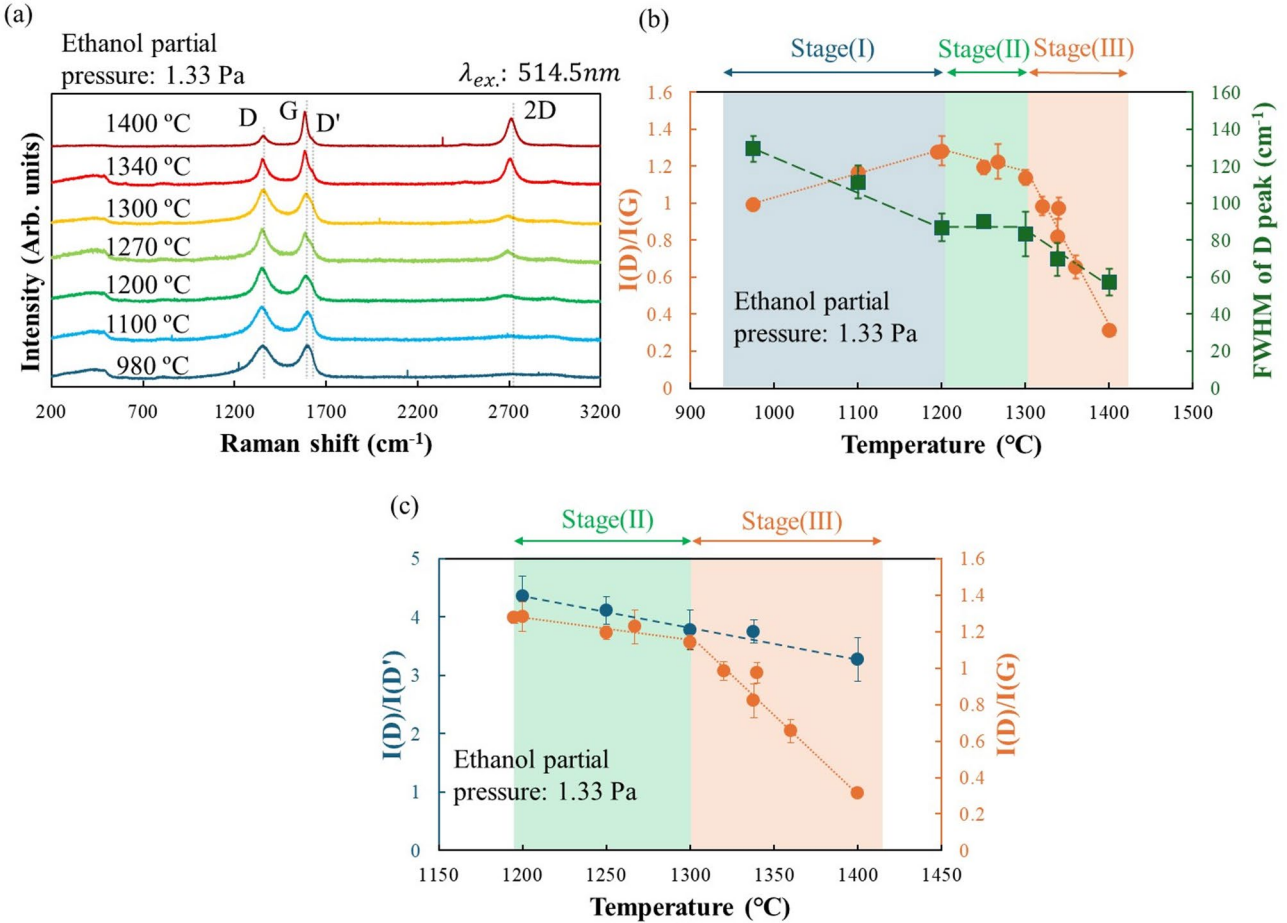
Evaluation of crystallinity based on Raman spectra of rGO thin films reduced at various temperatures in an ethanol atmosphere (ethanol partial pressure: 1.33 Pa; total pressure: 1.33 kPa). (**a**) Raman spectra at each reduction temperature. (**b**) Reduction temperature dependence of the intensity ratio I(D)/I(G) and the FWHM of the D peak $\:{{\Gamma\:}}_{D}$. (**c**) Reduction temperature dependence of the intensity ratio I(D)/I(D’) and I(D)/I(G). The temperature range is classified into three stages based on the changes in I(D)/I(G) and $\:{{\Gamma\:}}_{D}$.

Figure 1a shows typical Raman spectra of rGO thin films reduced at various temperatures in an ethanol atmosphere with a total pressure of 1.33 kPa (ethanol partial pressure: 1.33 Pa). The spectra exhibit peaks in the D band (~ 1350 cm⁻¹), G band (~ 1600 cm⁻¹), and 2D band (~ 2700 cm⁻¹) regions. Since the D peak originates from structural defects of graphene, the intensity ratio I(D)/I(G), calculated from the maximum intensities of the D and G peaks in the Raman spectrum, serves as an indicator of the crystallinity of graphene-based materials^24,26,29,30^.

Figure 1b shows the reduction temperature on the horizontal axis and both the intensity ratio I(D)/I(G) and the full width at half maximum (FWHM) of the D peak ($\:{{\Gamma\:}}_{D}$) as the vertical axis. To clearly distinguish the contributions to the spectral features, the peak intensity and FWHM were analyzed as independent parameters. $\:{{\Gamma\:}}_{D}$ is determined by fitting the D and G peaks with Lorentzian functions and extracting the full width at half maximum of the D peak (Supplementary Fig. S1). $\:{{\Gamma\:}}_{D}$ narrows as the *sp²* carbon network expands, which reflects improved structural ordering^26,31^.

Based on the variations in the intensity ratio I(D)/I(G) and $\:{{\Gamma\:}}_{D}$ with respect to the reduction temperature, as shown in Fig. 1b, the temperature ranges were classified into distinct stages. The temperature range where both an increase in the intensity ratio I(D)/I(G) and a narrowing of $\:{{\Gamma\:}}_{D}$ are observed is defined as Stage (I). The temperature range in which $\:{{\Gamma\:}}_{D}$ remains constant is defined as Stage (II). The temperature range where the intensity ratio I(D)/I(G) begins to decrease sharply and $\:{{\Gamma\:}}_{D}$ narrows again is defined as Stage (III).

The changes in the intensity ratio I(D)/I(G) and $\:{{\Gamma\:}}_{D}$ with increasing reduction temperature within the temperature ranges corresponding to Stage (I) and Stage (II) are consistent with previously reported trends^26^. It should be noted that the intensity ratio I(D)/I(G) exhibits non-monotonic behavior depending on the defect density in graphene^26,31–33^. Therefore, the crystallinity of rGO thin films cannot be evaluated solely based on the intensity ratio I(D)/I(G). However, complementary analysis of $\:{{\Gamma\:}}_{D}$ and *sp*^*2*^ C/*sp*^*3*^ C ratio (Supplementary Fig. S2), which reflect the degree of disorder in the *sp²* carbon network, clearly indicates that the crystallinity of the rGO thin films consistently improves from Stage (I) to Stage (III). Stage (I) is primarily associated with desorption of oxygen-containing functional groups from GO[^26^]. This desorption reveals the underlying *sp²* carbon network, thereby enhancing structural order and crystallinity (Supplementary Fig. S2). Since the D peak originates from *sp²* carbon network in proximity to structural defects, the exposure of the *sp²* carbon network contributes to an increase in D peak intensity^32^. Consequently, the intensity ratio I(D)/I(G) increases and $\:{{\Gamma\:}}_{D}$ gradually narrows as the reduction temperature rises.

Stage (II) is characterized by the repair of vacancies in rGO through the adsorption of carbon precursors onto dangling bonds^24–27,29^. This repair process facilitates lateral expansion of the *sp²* carbon network and results in a lower defect density compared to Stage (I). In this stage, the crystallinity of rGO, as assessed by the intensity ratio I(D)/I(G), increases only marginally, and the change is sufficiently small that $\:{{\Gamma\:}}_{D}$ remains nearly constant (Fig. 1b). At higher reduction temperatures corresponding to Stage (III), a pronounced decrease in the intensity ratio I(D)/I(G) and further narrowing of $\:{{\Gamma\:}}_{D}$ are observed. The significant crystallization occurring in Stage (III) is clearly distinct from the trends observed in Stages (I) and (II), suggesting the involvement of a previously unreported crystallization mechanism (Supplementary Fig. S3).

## Estimation of defect structures

The reduction of GO at the temperatures corresponding to Stage (III) in an ethanol atmosphere was found to markedly enhance the crystallinity of rGO. To clarify the crystallization mechanism operative in Stage (III), the evolution of defect structures associated with this pronounced crystallization behavior was analyzed.

Raman spectra can be deconvoluted into multiple peaks (Supplementary Fig. S1). Structural defects in graphene typically include *sp*³-defects, vacancies, and grain boundaries. Since the D’ peak is highly sensitive to the type of defect, the intensity ratio I(D)/I(D’) between the D and D’ peaks can be used to infer the nature of the defects^26,30,34,35^. The intensity ratio I(D)/I(D’) corresponds to structural defects of *sp*³-defects (~ 13), vacancies (~ 7), and grain boundaries (~ 3.5), respectively^35^. Figure 1c shows the intensity ratio I(D)/I(D’) and I(D)/I(G) (same as Fig. 1b) in the temperature range from Stage (II) to Stage (III). The intensity ratio I(D)/I(D’) is observed to transition linearly from Stage (II) to Stage (III). Fitting results for each of the peaks yielded I(D)/I(D’) ≈ 4.4 at 1200 ℃ and I(D)/I(D’) ≈ 3.3 at 1400 ℃ (Supplementary Fig. S1). Therefore, the structural defects of rGO are suggested to change from a coexistence of grain boundaries and minor vacancies to predominantly grain boundaries as the reduction temperature increases from Stage (II) to Stage (III). This is consistent with previously reported models of rGO crystallization, in which the repair of vacancies progresses through the adsorption of carbon precursors onto dangling bonds within rGO^21–27^. In contrast, a pronounced decrease in the intensity ratio I(D)/I(G) is observed at Stage (III). This suggests that the dramatic crystallization observed at Stage (III) involves a mechanism distinct from defect repair in rGO.

## Change in surface morphology of rGO thin films as a function of ethanol partial pressure

Analysis of defect structures based on Raman spectroscopy indicates that mechanisms beyond the simple repair of vacancies contribute to the pronounced crystallization observed in Stage (III). Previous studies have reported that high-temperature thermal reduction in an ethanol atmosphere leads to the formation of carbon aggregates on the surface of rGO^27,29^. To clarify the crystallization mechanism operative in Stage (III), we investigated the influence of these carbon aggregates on the surface morphology and crystallinity of rGO thin films.

**Fig. 2:**
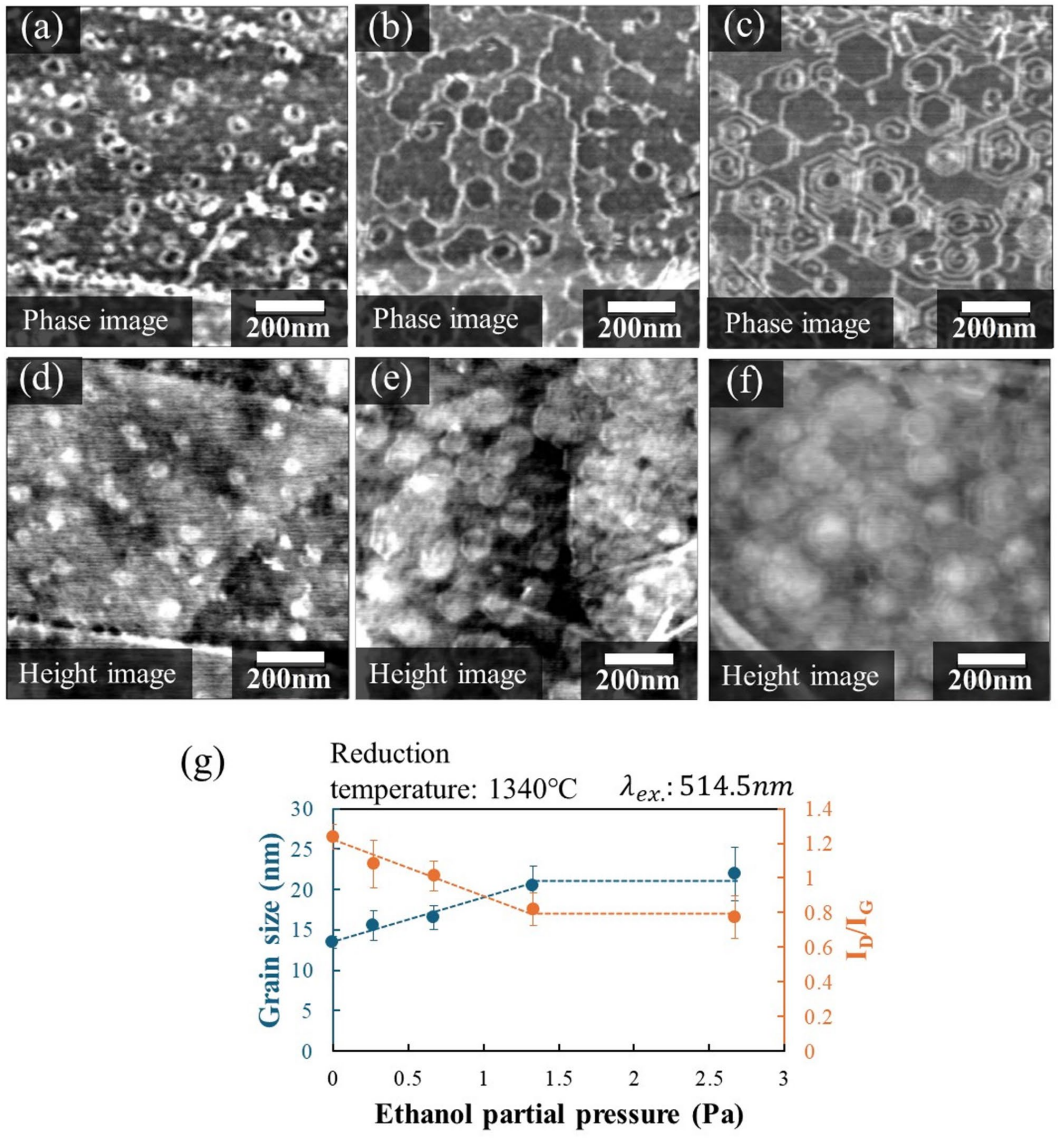
AFM phase and height images of the rGO surfaces thermally annealed at 1340 °C in an ethanol atmosphere, with varying ethanol partial pressures: (**a**, **d**) 0.27 Pa, (**b**, **e**) 0.67 Pa and (**c**, **f**) 2.67 Pa, respectively. (**g**) Ethanol partial pressure dependence of the intensity ratio I(D)/I(G) and grain size.

Figure 2a–f show the atomic force microscopy (AFM) phase and height images of the rGO surface thermally reduced at 1340 °C under varying ethanol partial pressures. At a low partial pressure of 0.27 Pa (Fig. 2a, d), small amorphous carbon aggregates originating from carbon precursors are observed. At an intermediate partial pressure of 0.67 Pa (Fig. 2b, e), two‑dimensional island structures emerge. At a higher partial pressure of 2.67 Pa (Fig. 2c, f), the islands further increase in lateral size and exhibit multilayered structures with hexagonal edges. These edge morphologies reflect crystallographic orientations associated with slower growth kinetics; among graphene edge structures, the zigzag edges exhibit the slowest growth rate^36^. Consequently, the graphene islands preferentially adopt hexagonal shapes. Figure 2g summarizes the variation in the intensity ratio I(D)/I(G) and the grain size of rGO as a function of ethanol partial pressure during thermal reduction at 1340 °C in an ethanol atmosphere. The grain size $\:{L}_{a}$ is evaluated using Eq. (1)^37^.

$$\:\begin{array}{c}{L}_{a}\left[nm\right]=\frac{560}{{{E}_{l}}^{4}}{\left(\frac{I\left(D\right)}{I\left(G\right)}\right)}^{-1}\#\left(1\right)\end{array}$$

As shown in Fig. 2g, the crystallinity of rGO increases with increasing ethanol partial pressure. Here, it should be noted that \:{L}_{a} reflects the average grain size of the entire rGO thin film and does not represent the size of individual graphene islands. Using the $\:{L}_{a}$ value obtained under an inert atmosphere (ethanol partial pressure: 0 Pa; ~14 nm) as a reference, and given that the morphology of the rGO surface is significantly altered by the introduction of a carbonaceous gas, the observed enhancement is attributed to the formation of graphene islands on the rGO surface during thermal reduction in an ethanol atmosphere. However, when the ethanol partial pressure exceeds 1.33 Pa, the improvement in crystallinity gradually saturates, and asymptotically approaches a constant value.

## Evaluation of surface morphology of rGO thin films as a function of reduction temperature

**Fig. 3:**
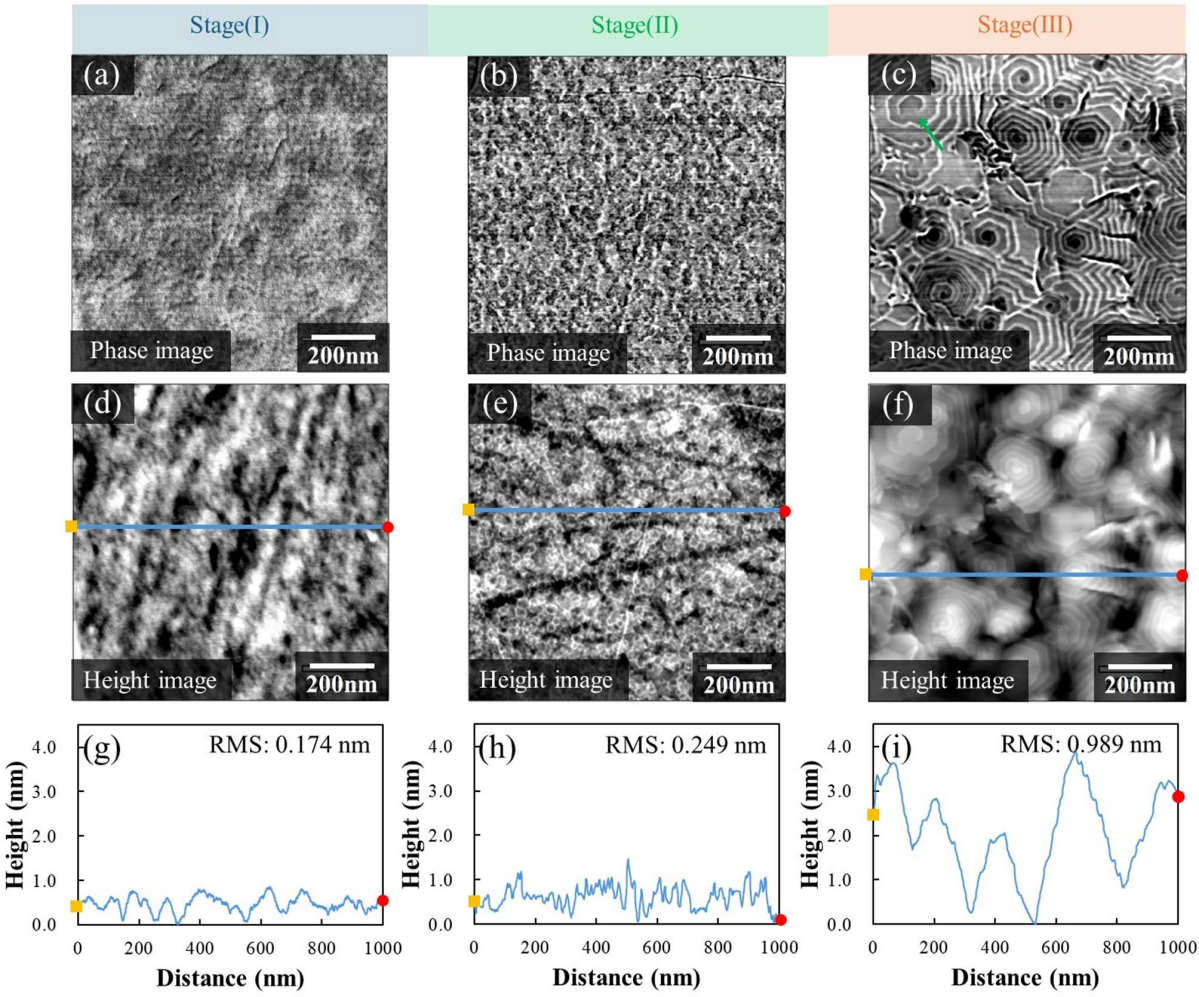
AFM phase and height images of the rGO surface after thermal reduction in an ethanol atmosphere (ethanol partial pressure: 1.33 Pa) at (**a**, **d**) 1100 °C (Stage (I)), (**b**, **e**) 1250 °C (Stage (II)), and (**c**, **f**) 1400 °C (Stage (III)). (**g**–**i**) Corresponding height profiles extracted along the blue lines obtained from the AFM height images. The RMS surface roughness values were evaluated from height profiles shown in (**d**–**f**).

Figure 3a–f show the AFM phase and height images of the rGO surface after thermal reduction at 1100 °C (Stage (I)), 1250 °C (Stage (II)), and 1400 °C (Stage (III)) in an ethanol atmosphere with a partial pressure of 1.33 Pa. At Stage (I), no ethanol-derived carbon aggregates are observed on the rGO surface (Fig. 3a, d). In contrast, at Stage (II), minute carbon aggregates are uniformly formed across the surface (Fig. 3b, e). At the higher temperature of Stage (III), multilayered islands with hexagonal edges appear and are widely distributed over the rGO surface (Fig. 3c, f). Figure 3g–i show the height profiles along the blue lines in Fig. 3d–f obtained from AFM height images. The value of RMS (Root Mean Square) surface roughness at Stage (I) is 0.174 nm, which is comparable to that of rGO reduced in an argon atmosphere. At Stage (II), the value of RMS surface roughness slightly increases to 0.249 nm, likely due to the formation of small carbon aggregates. In contrast, Stage (III) exhibits a significant increase in RMS surface roughness to 0.989 nm. This increase is attributed to the multilayered islands, featuring pyramid-like three-dimensional structures with heights of several nanometers. As highlighted by the green arrows in Fig. 3c, part of the uppermost island layer appears to be bonded to the underlying layer. The detailed formation mechanism of these multilayered islands is discussed later.

**Fig. 4:**
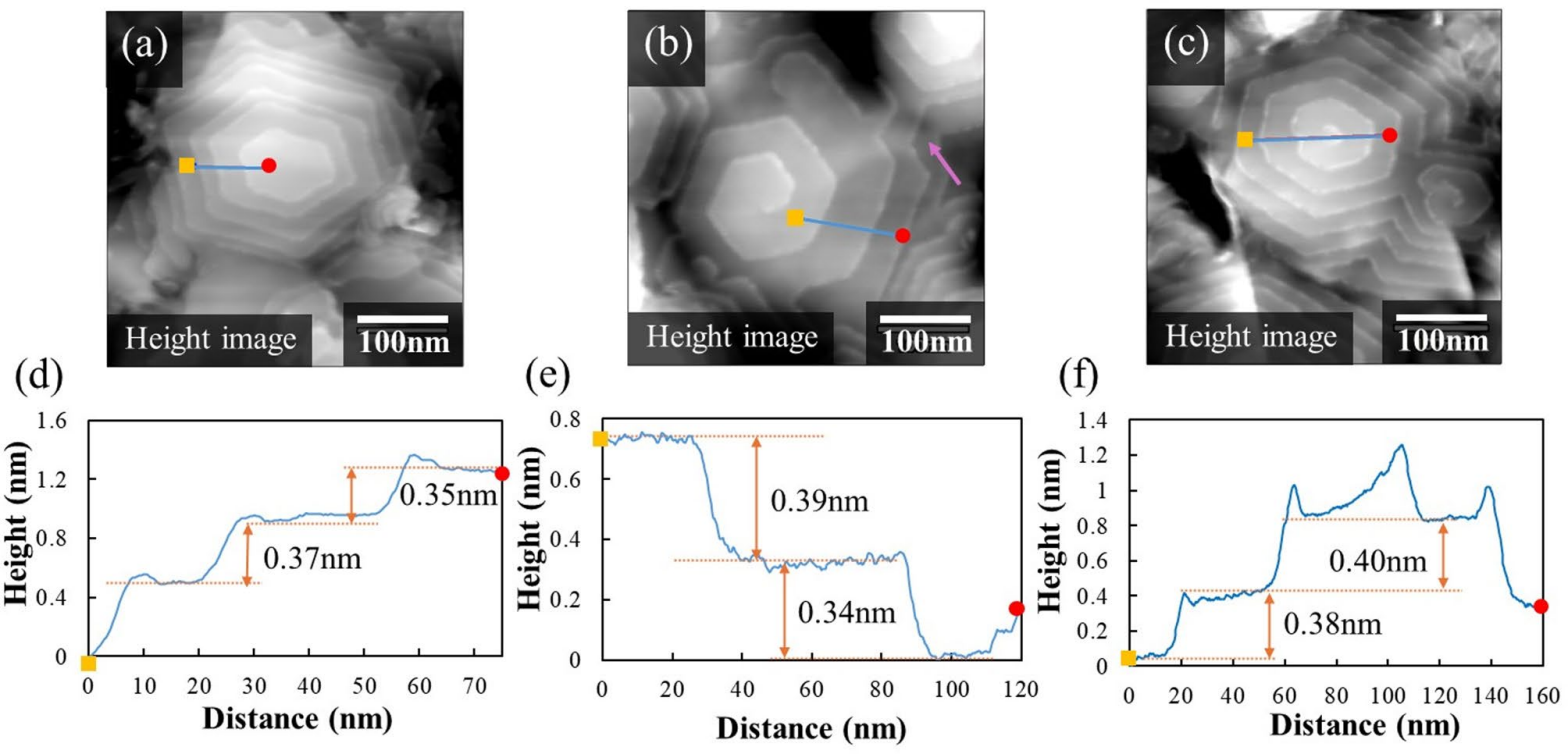
AFM height images of multilayered graphene islands with (**a**) a layer-by-layer structure and (**b**, **c**) a spiral structure. The multilayered graphene islands with a spiral structure result from (**b**) a screw dislocation and (**c**) a Frank–Read source. The corresponding height profiles along the blue lines are shown below each image in (**d**–**f**). The purple arrow in (**b**) indicates a smoothly merged boundary between adjacent island edges.

In the temperature range corresponding to Stage (III), where a significant enhancement in crystallinity is observed, three-dimensional structures are observed to form on the rGO surface as shown in Fig. 3c, f. Figure 4 shows the AFM height images and corresponding height profiles of multilayered islands on the rGO surface thermally reduced at 1400 °C (Stage III)). The multilayered islands can be classified into two types: layer-by-layer structures (Fig. 4a) and spiral structures (Fig. 4b and c). The multilayered islands shown in Fig. 4a are attributed to the homoepitaxial growth of graphene, proceeding via layer-by-layer growth in which each monolayer is sequentially stacked uniformly on the rGO surface, forming distinct step edges. As shown in the height profile in Fig. 4d, steps with monolayer height are clearly observed. These observations indicate that the crystal growth process yields a highly crystalline structure with excellent lattice matching and no discernible structural defects such as dislocations.

By contrast, the island with the spiral structure shown in Fig. 4b is similar to the graphene spirals reported in previous studies^38,39^. This structure is thought to result from screw dislocations formed at edges or defect sites on the rGO surface, which act as nucleation sites and inducing spiral growth. The spiral structure exhibits a characteristic geometry in which each layer is continuously connected along the out-of-plane direction. The multilayered islands shown in Fig. 4c exhibits a similar feature, where a portion of the uppermost layer is continuously connected to the underlying layer, as also observed in the multilayered island indicated by the green arrow in Fig. 3c. This morphology is attributed to spiral growth induced by a dislocation originating from a Frank–Read source. A Frank–Read source consists of paired right- and left-handed screw dislocations^39–41^. As these two screw dislocations propagate in mutually outward rotational directions, multilayered islands, as observed in Fig. 4c, are formed. Accordingly, this structure is classified as a spiral structure. The geometrical features of the graphene islands directly observed by AFM indicate the presence of dislocations, such as screw dislocations and Frank–Read sources. The island morphologies associated with such dislocations are consistent with those reported in other material systems, including WS_2_^42^, SnS^43^, and nematic liquid crystals of 4-cyano-4’-pentylbiphenyl^40^. The height profiles shown in Fig. 4d–f and the X-ray diffraction (XRD) measurements (Supplementary Fig. S4 and Table S1), consistently indicate that the interlayer spacing of the multilayered graphene islands is slightly larger than, yet in good agreement with, the interlayer spacing of 0.335 nm typically observed in AB-stacked graphene. As shown in Figs. 2c, f, 3c, f, and 4a–c, the graphene islands are aligned along a common orientation, suggesting that this alignment originates from interlayer interactions between the rGO template and the growing graphene islands. Furthermore, the area indicated by the purple arrow in Fig. 4b shows where adjacent islands are connected, appearing to exhibit smoothly merged boundaries between island edges. These observations indicate that the lateral expansion of graphene islands occurs not only through layer-by-layer growth but also via the coalescence of independently grown islands originating from multiple nucleation sites. This process facilitates the formation of a highly crystalline rGO thin film with minimal structural defects.

## Hall effect measurement of rGO thin films

**Fig. 5:**
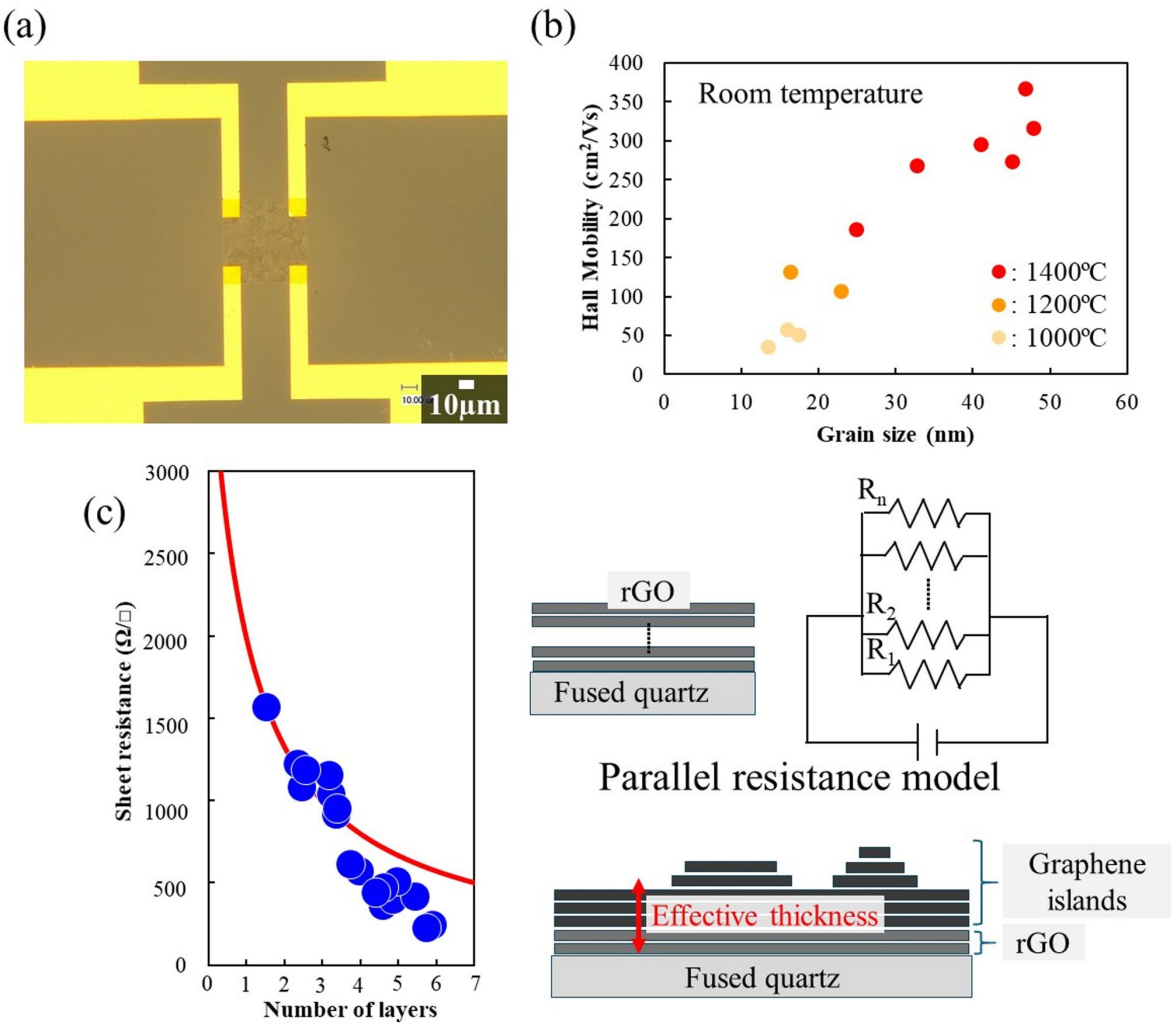
(**a**) Correlation between the grain size of rGO thin films, estimated from Raman spectra, and carrier mobility measured by the van der Pauw method. (**b**) Optical micrograph of an rGO thin film device for Hall effect measurements based on the van der Pauw method. (**c**) Dependence of sheet resistance on the number of layers in the rGO thin films. The solid red line represents the theoretical sheet resistance calculated based on a parallel resistance model, as illustrated on the right side of the graph.

An optical micrograph of the rGO device used for Hall effect measurements based on the Van der Pauw method is shown in Fig. 5a. The rGO thin film forms a continuous and uniform conducting sheet, with four ohmic contacts positioned at the periphery of the channel. Hall measurements performed in this configuration exhibit a symmetry factor of ~ 1. This empirical evidence supports the assumption of macroscopic uniformity in electrical conductivity, thereby justifying the application of the Van der Pauw method despite the local microscopic inhomogeneity.

Figure 5b shows the relationship between the grain size of the rGO thin film, evaluated from Raman spectra, and the Hall mobility measured using the Van der Pauw method. The grain size is controlled by varying the reduction temperature under constant gas-phase conditions. As the grain size increases, indicating improved crystallinity, the Hall mobility increases linearly and reaches a maximum of 365 cm²/V·s at room temperature. A previous study reported a record-high mobility of ~ 320 cm²/V·s for rGO thin films obtained by thermal reduction of GO^15^. Therefore, the mobility achieved in this study represents one of the highest values reported to date for rGO thin films.

Figure 5c shows the variation in sheet resistance as a function of the number of layers in rGO thin films with graphene islands. The number of layers is controlled by varying the growth time, while maintaining the GO thin film at 1–3 layers^26^ and keeping the gas-phase conditions constant. The number of layers is evaluated using the intensity ratio I(G)/I(sub.) between the G band peak and the substrate-derived peak from fused quartz at ~ 460 cm⁻¹ in the Raman spectra^44^. The solid red line represents the theoretical sheet resistance calculated based on a parallel resistance model, as illustrated in the upper-right side of the graph, using the sheet resistance of rGO thin films without graphene islands as a reference. Because the graphene islands exhibit pyramid-like multilayered structures distributed throughout the entire channel, the Raman signal represents a spatially averaged response. Accordingly, non-integer layer numbers are interpreted as effective thickness over the channel region, as illustrated in the lower-right side of the graph For films with five or more layers, the measured sheet resistance deviates from the theoretical curve and exhibits lower values. While the sheet resistance decreases significantly with increasing layer number, the increase in carrier density is relatively modest (Supplementary Fig. S7). Despite the increase in film thickness (number of layers), the carrier density per unit thin film remained almost constant. This suggests that the observed reduction in sheet resistance is attributable to the stacking of graphene islands, which possess higher crystallinity and fewer charged impurities compared to the original rGO template.

## Discussion

**Fig. 6:**
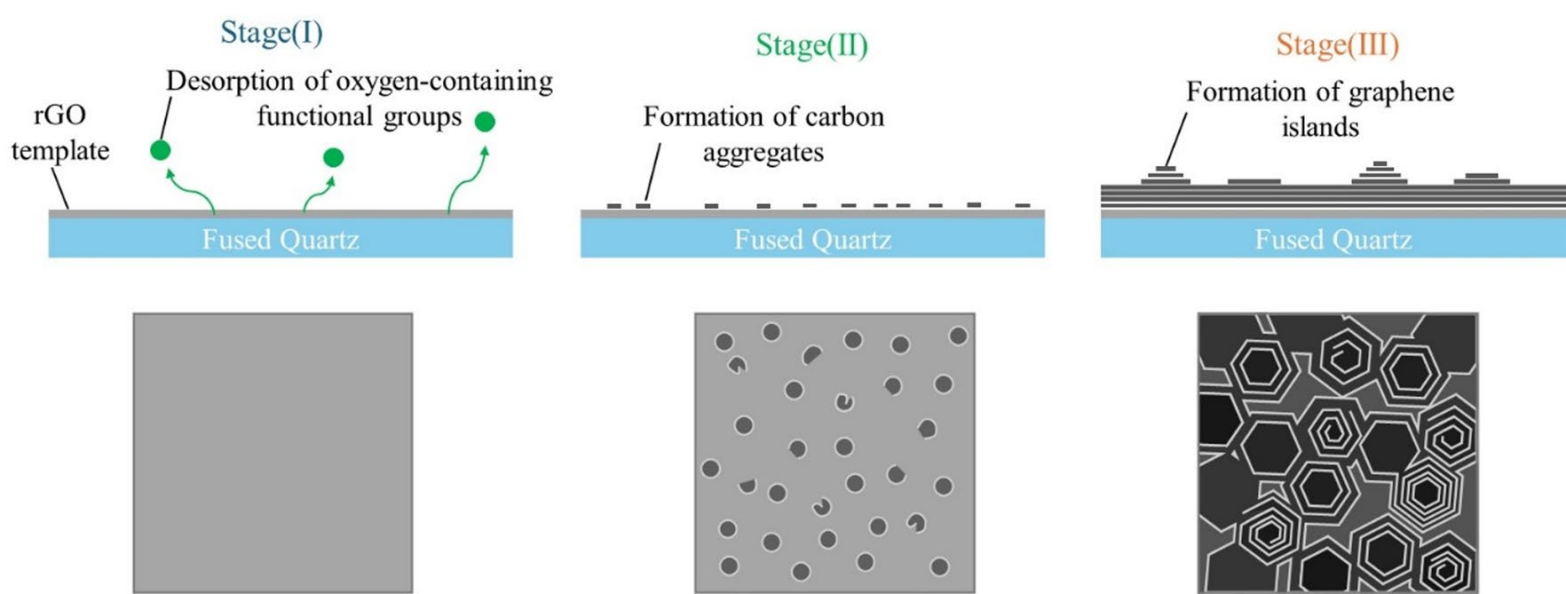
Schematic illustration of the crystallization mechanisms of rGO thin films at each stage of the thermal reduction in an ethanol atmosphere.

Figure 6 shows a schematic illustration of the crystallization mechanisms of rGO thin films across different temperature ranges. In Stage (I), the thermal energy supplied during the reduction process promotes the desorption of oxygen-containing functional groups, leading to progressive crystallization. However, due to the relatively low temperature, the driving force for graphene growth remains insufficient, and the generation of carbon precursors is negligible.

In Stage (II), vacancies are repaired as carbon precursors adsorb onto the dangling bonds present in rGO. Meanwhile, it has also been reported that carbon precursors can adsorb and aggregate on the rGO surface, leading to the formation of carbon aggregates^27,29^. These adsorbed carbon aggregates lack sufficient activation energy to undergo structural reorganization into a stable *sp²* carbon network, resulting instead in the formation of an amorphous-like structure containing *sp*³-defects.

In Stage (III), the high-temperature conditions provide sufficient activation energy for structural reorganization, allowing graphene islands composed of *sp²* carbon networks to crystallize on the rGO surface, originating from carbon precursors. Two types of multilayer graphene islands are observed in this stage: layer-by-layer structures and spiral structures. Both types exhibit well-defined step features corresponding to the thickness of monolayer graphene.

Structural analysis using Raman spectroscopy and AFM imaging at the reduction temperature corresponding to Stage (III) revealed a significant improvement in the crystallinity of the rGO thin film. This improvement is believed to be attributable to the crystalline growth of graphene islands on the rGO template. Indeed, in our previous study^44,45^, we demonstrated that graphene grows homoepitaxially on a graphene template under vapor-phase conditions similar to those in this study (see Supplementary Fig. S6). Based on these findings, they suggest that graphene layers grow homoepitaxially on the rGO template via a similar mechanism, and the resulting graphene islands possess higher crystallinity than the underlying template itself. Indeed, the 002 diffraction peaks obtained from XRD measurements show that the graphene islands exhibit a sharper peak than the rGO template, confirming their superior crystallinity (Supplementary Fig. S4 and Table S1).

This result, indicating that the graphene islands exhibit higher crystallinity than the rGO template, is in good agreement with the observed dependence of sheet resistance on the number of layers. This implies that the *sp²* carbon network of graphene in the growth plane extends laterally beyond the grain boundaries present in the rGO template. The tendency for the upper epitaxially grown layers to possess higher crystallinity than the underlying template is considered a characteristic feature of van der Waals epitaxial growth in layered materials. This enhanced crystallinity of the grown layers is attributed to the π‑bonding interlayer interactions along the c-axis direction of graphene.

The 2D band in the Raman spectrum of multilayer graphene is known to be highly sensitive to interlayer interactions^46,47^. In this study, analysis of the 2D peak reveals a turbostratic stacking ratio $\:R{\prime\:}$ of approximately 63.4% (Supplementary Fig. S5). This ratio suggests that a perfectly AB-stacked structure is not formed in the rGO thin film during the crystal growth of graphene islands. As a result, the graphene islands are not strongly constrained by the crystallinity of the underlying template, enabling them to achieve high crystallinity.

**Fig. 7:**
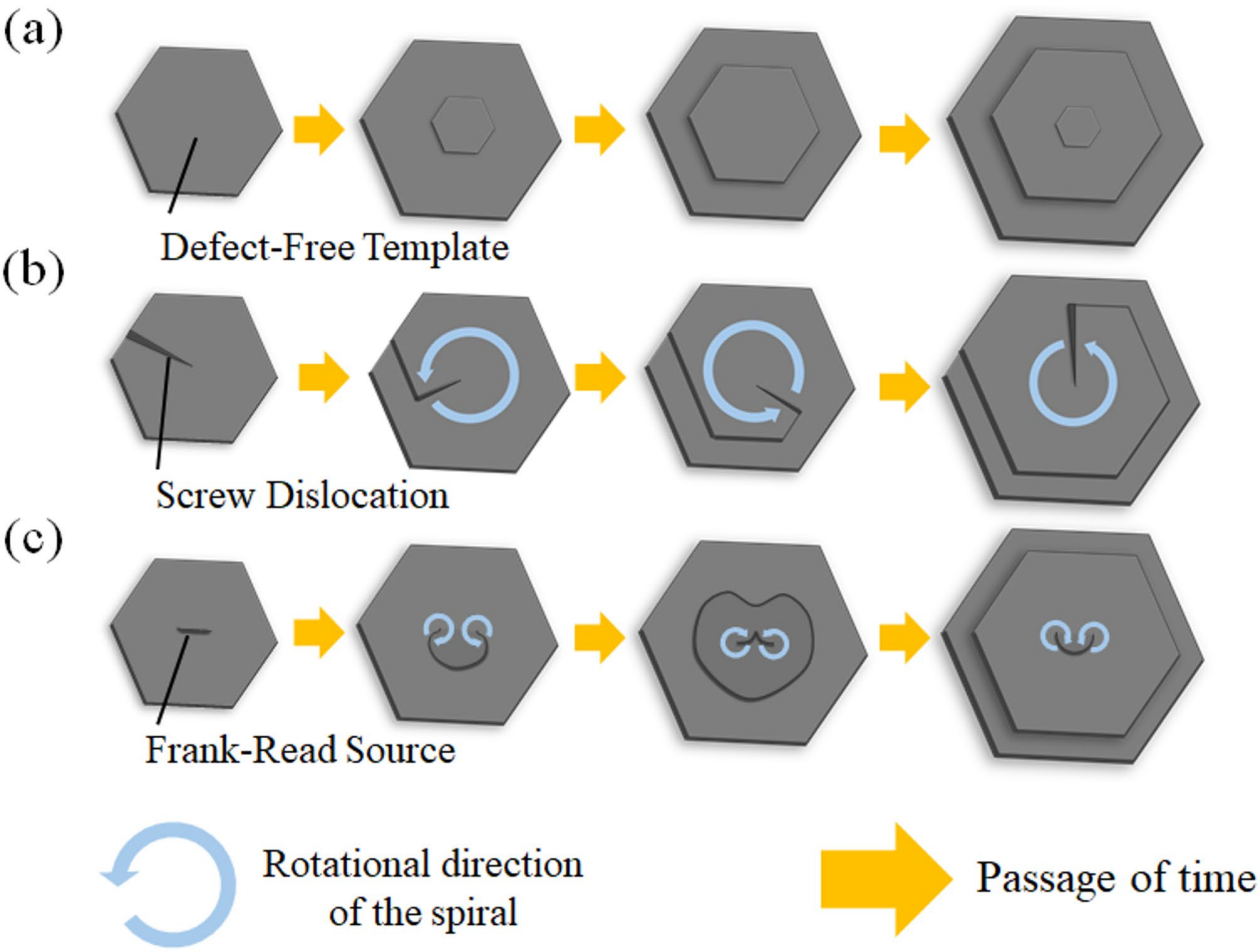
Schematic illustration of the crystal growth processes of graphene islands via two distinct growth modes: (**a**) layer-by-layer growth and (**b**, **c**) spiral growth. Spiral growth originates from (**b**) a screw dislocation and (**c**) a Frank–Read source.

Figure 7 illustrates the crystallization processes of graphene islands via two distinct crystal growth modes: layer-by-layer growth and spiral growth. These differences in behavior are attributed to variations in the crystal structure of the rGO template, which serves as a nucleation site for the carbon precursor. In the absence of structural defects such as dislocations (Fig. 7a), nucleation of graphene is followed by epitaxial growth, resulting in the formation of islands with a layer-by-layer structure. In contrast, when a screw dislocation is present in the rGO template (Fig. 7b), the carbon precursors are adsorbed at the dislocation, which serves as a preferential nucleation site, leading to the development of the spiral structure. Furthermore, when the template contains a dislocation originating from a Frank–Read source (Fig. 7c), a geometric feature characteristic of the Frank–Read mechanism is observed in the uppermost layer of the resulting graphene islands. These islands also exhibit continuously connected spiral structures, analogous to those formed via spiral growth, indicating that the *sp²* carbon network is preserved throughout the vertical growth process. Importantly, regardless of the crystal growth mode, in-plane crystallization proceeds through the homoepitaxial growth mechanism, enabling the formation of an extended *sp²* carbon network with minimal defects. These findings indicate that, although the crystal growth modes are governed by the structure of the underlying template, highly crystalline graphene islands can be grown on rGO templates.

## Summary

In this study, we systematically investigated the crystallization mechanisms of rGO thin films during thermal reduction in an ethanol atmosphere, with particular emphasis on the effects of reduction temperature and ethanol partial pressure on the structural, morphological, and electrical properties of rGO. Comprehensive characterization using Raman spectroscopy, AFM, XRD, and electrical transport measurements revealed a multi‑stage evolution of crystallinity and surface morphology. Notably, we identified a previously unreported crystallization mechanism dominated by the homoepitaxial growth of graphene islands on the rGO surface. These findings provide fundamental insights into the structural transformation of GO through thermal reduction in an ethanol atmosphere and suggest a viable strategy for the scalable and controllable fabrication of high-crystallinity rGO thin films with superior electronic properties.

## Methods

### Preparation of GO thin films

GO was synthesized following previously reported methods^2^. GO thin films were fabricated using an electrostatic self-assembly approach^26,48^. A fused silica substrate (Shin-Etsu Chemical) was first immersed for 1 h in a solution of 3-aminopropyltrimethoxysilane (APTMS, purity > 96.0%, Tokyo Chemical Industry) and ethanol mixed at a volume ratio of 1:9, thereby forming an amino-terminated self-assembled monolayer (SAM) on the substrate surface. After annealing the SAM-modified substrate at 120 °C for 30 min on a hot plate, the substrate was immersed overnight in a 0.003 wt% aqueous GO dispersion, resulting in the formation of GO thin films composed of one to three layers.

### Thermal reduction of GO thin films

The reduction of GO was carried out using a custom-built cold-wall infrared heating furnace. Unlike conventional hot-wall furnaces, the cold-wall configuration enables localized heating of only the substrate, thereby allowing reduction at elevated temperatures. This design also suppresses undesired heating of the surrounding chamber, effectively inhibiting secondary gas-phase association reactions of ethanol decomposition products and preventing the deposition of carbon aggregates on the rGO surface. Argon, an inert gas, was employed as the carrier gas, while ethanol served as the carbonaceous. The flow rates of all gases were precisely controlled using mass flow controllers. The temperature ramp rate was set to 100 °C/min up to 1000 °C, and 50 °C/min up to 1400 °C. Upon reaching the target temperature, the total chamber pressure was maintained at 1.33 kPa. During the reduction process, the Ar flow rate was kept constant at 500 sccm, while the ethanol flow rate was varied between 0 and 3 sccm depending on the experimental conditions.

### Characterization of rGO Thin Films

To investigate the surface morphology of the rGO thin film, atomic force microscopy (AFM; Jupiter, Oxford Instruments) was performed in AC mode (tapping mode). The crystallinity of the rGO thin film was evaluated by measuring Raman spectra using a micro-Raman spectrometer (LabRAM HR-800, HORIBA Jobin Yvon). Raman measurements were carried out at room temperature with a 100× objective lens and a laser excitation wavelength of 514.5 nm. The chemical bonding states of the rGO films were analyzed using X-ray photoelectron spectroscopy (XPS; PHI Quante, Ulvac PHI) with a monochromatic Al Kα X-ray source (1486.6 eV). In addition, θ-2θ scans were conducted using an X-ray diffractometer (XRD; SmartLab, Rigaku) to assess the stacking structure of the rGO thin films. Measurements were performed over a 2θ range of 5°-60° with a step size of 0.02°, using a monochromatic Cu Kα radiation source (λ = 0.154 nm).

### Transistor fabrication and Electrical transport measurements

The carrier mobility and sheet resistance of the rGO thin films were determined by Hall effect measurements using the van der Pauw configuration with a Bio-Rad HL5500PC system. Device fabrication was carried out using photolithography (Mask-less Exposure System; DL-1000/NC2P, NanoSystemSolutions) followed by electron beam lithography (ELS-G125, Elionix). The channel was defined by reactive ion etching (RIE; NE-550, ULVAC). Metal electrodes (Au/Ti, 80/10 nm) were deposited by electron beam evaporation (EX-550, ULVAC). All measurements were performed at room temperature under ambient conditions.

## Supplementary Information

Below is the link to the electronic supplementary material.

Supplementary Material 1

## Data Availability

All data measured during and/or analyzed during the current study are available from the corresponding author on reasonable request.
