# Supplementary material for: Enhanced crystallinity and electrical properties through homoepitaxial growth on reduced graphene oxide templates
Source: Sci Rep. 2026 May 30;16:24766. doi: 10.1038/s41598-026-54930-1 (PMC13458042; doi:10.1038/s41598-026-54930-1)
Supplement: Supplementary file 1 [file 41598_2026_54930_MOESM1_ESM.docx]

**Supplementary figures**

Title:

**Enhanced Crystallinity and Electrical Properties through Homoepitaxial Growth on Reduced Graphene Oxide Templates**

S. Kanda^1^*, T. Yamashita^1^, S. Kurosu^2^, F. Sakamoto^2^, T. Hanajiri^1,2^, Y. Nishina^3^ & R. Negishi^1, 2^

*^1^Graduate School of Science and Engineering, Toyo Univ., 2100 Kujirai, Kawagoe-city, Saitama 350-8585, Japan*

*^2^Bio-nanoelectronics Research Centre, 2100 Kujirai, Kawagoe-city, Saitama 350-8585, Japan*

*^3^Okayama Univ., 3-1-1 Tsushimanaka, Kita-ku, Okayama-city, Okayama 700-8530, Japan*

*Corresponding author: Tel: +81-49-239-1366

e-mail: s36C02400048@toyo.jp (S. Kanda), [negishi046@toyo.jp](mailto:negishi046@toyo.jp) (R. Negishi)

**Peak fitting of Raman spectra of rGO thin films**

Figure S1 shows the results of peak fitting for the Raman spectra of rGO reduced at 1400 °C (Stage (III)) and 1250 °C (Stage (II)) in an ethanol atmosphere (ethanol partial pressure: 1.33 Pa). The Raman spectra of rGO reduced at Stage (III) were fitted with three peaks: D', G, and D, as shown in Fig. S1(a). In contrast, the Raman spectra of rGO reduced at Stage (II) were fitted with five peaks: D', G, D, and two additional broad peaks of D'' (1500–1550 cm⁻¹) and D* (1150–1200 cm⁻¹), as shown in Fig. S1(b). All peaks were fitted with Lorentzian functions. To simplify the fitting, the D' peak was fixed at 1620 cm⁻¹. The D'' peak is associated with amorphous carbon, and its peak intensity correlates with crystallinity^1,2^. The D* peak originates from lattice vibrations of graphene with sp³-defects induced by oxygen-containing functional groups^3^.

The Raman spectra of rGO at Stage (II) (Fig. S1(b)) exhibit the D'' and D* peaks. In contrast, these two peaks are absent in the Raman spectra of rGO at Stage (III) (Fig. S1(a)). This indicates that high-temperature thermal reduction in an ethanol atmosphere increases the proportion of the sp² carbon network within the rGO thin film.

**Figure S1|** Peak fitting of the Raman spectra of rGO thin films reduced at (a) 1400 °C (Stage (III)) and (b) 1250 °C (Stage (II)).

**Analysis of chemical bonding states**

To analyze the chemical bonds in rGO, quantitative analysis was conducted using X-ray photoelectron spectroscopy (XPS). The C 1s spectrum is characterized by the following components: *sp²* C=C (~284.3 eV) originating from graphitic six-membered carbon rings; *sp³* C-C (~284.8 eV) originating from hydrocarbons and amorphous carbon; C–O (~286.1 eV); C–O–C (~287.1 eV); C=O (~288.4 eV); and O=C–O (~289.5 eV), which are attributed to oxygen-containing functional groups. In addition, a π–π* (291.1–291.4 eV) satellite peak appears, originating from the π electrons of graphene^4^.

Figure S2(a) shows the C 1s spectrum of GO before thermal reduction. Peaks originating from various oxygen-containing functional groups are observed, while the *sp²* C=C peak is absent. Figures S2(b–f) present the C 1s spectra of rGO reduced at each temperature in an ethanol atmosphere (ethanol partial pressure: 1.33 Pa). With increasing reduction temperature from 1000 °C (Stage (I)) to 1400 °C (Stage (III)), the peaks associated with oxygen-containing functional groups are significantly attenuated, whereas the *sp²* C=C peak becomes clearly pronounced. To evaluate the reduction degree (R. D.) from the C 1s spectra, equation (S1) was defined based on the relative percentage of each chemical-bond component.

$$\begin{aligned} R. D. \left( \% \right)=\left( {sp}^{2} C=C \right)+\left( {sp}^{3} C-C \right)+\left( \pi-\pi^{*} \right)\#\left( S1 \right) \end{aligned}$$

Figure S2(g) shows the reduction degree at each reduction temperature. The R. D. values at 1000 °C (Stage (I)), 1250 °C (Stage (II)), and 1400 °C (Stage (III)) are 84.4 %, 86.7 %, and 87.3 %, respectively. In contrast, the R. D. of GO is 48.5 %, indicating the effective desorption of oxygen-containing functional groups during thermal reduction in an ethanol atmosphere. Figure S2(h) presents the ratio of *sp²* C=C to sp³ C–C (${sp^{2} C}/{sp^{3} C}$) at each reduction temperature. The upward trend with increasing reduction temperature indicates an increase in the proportion of *sp²* C=C bonds of rGO thin films. These results suggest that the graphene islands epitaxially grown on the rGO template possess higher crystallinity than the template itself, consistent with the Raman spectroscopy results.

**Figure S2|** (a) XPS spectrum of GO and (b–f) XPS spectra of rGO reduced at each reduction temperature. (g) Reduction degree of rGO at each reduction temperature. (h) Ratio of sp² C to sp³ C (sp² C/sp³ C) for rGO reduced at each reduction temperature.

**Thermal reduction in an inert argon atmosphere**

To demonstrate the effect of the ethanol atmosphere on crystallization, thermal reduction was performed in an inert argon atmosphere at a total pressure of 1.33 kPa. Figure S3 shows the intensity ratio I(D)/I(G) at each reduction temperature. The blue dots represent the intensity ratio I(D)/I(G) of rGO reduced in an argon atmosphere. For comparison, the orange dots indicate the intensity ratio I(D)/I(G) of rGO reduced in an ethanol atmosphere (the same data as in Fig. 1(b)). In an inert atmosphere, the intensity ratio I(D)/I(G) remains almost constant in the temperature range of 1100–1400 °C. This indicates that the dramatic crystallization of rGO observed at Stage (III) in an ethanol atmosphere is not attributable to heat-induced reconstruction of the crystal structure (graphitization^5,6^).

**Figure S3|** The intensity ratio I(D)/I(G) of rGO reduced in an ethanol (orange) and an argon (blue) atmosphere.

**Assessment of stacking structures**

To assess the stacking structure of the graphene islands formed on rGO, X-ray diffraction (XRD) measurements were performed. Figure S4(a) shows the XRD pattern of an rGO thin film reduced in an argon atmosphere (ethanol partial pressure: 0 Pa). Because ethanol was not introduced during thermal reduction in the argon atmosphere, no graphene islands were formed on the rGO surface. As shown in Fig. S4(a), a broad 002 diffraction peak^7^ originating from the rGO template is observed. Figure S4(b) presents the XRD pattern of an rGO thin film reduced in an ethanol atmosphere. As in Fig. 4(c), thermal reduction at 1400 °C (Stage (III)) in the ethanol atmosphere leads to the formation of graphene islands on the rGO surface. In addition to the broad 002 peak, a sharp 002 diffraction peak^8^ is clearly observed in Fig. S4(b). This sharp peak appears only when thermal reduction is carried out in the ethanol atmosphere and is therefore attributed to the formation of graphene islands. The significantly sharper 002 diffraction peak of the graphene islands compared to that of the rGO template indicates that the graphene islands possess higher crystallinity. These results are consistent with the Raman spectroscopy and XPS results.

Equation (S2) shows Bragg's equation.

$$\begin{aligned} n\lambda=2d\sin\theta\#\left( S2 \right) \end{aligned}$$

According to equation (S2), the interlayer spacing $d_{002}$ of the epitaxially grown graphene islands is 0.340 nm (Table S1), which is slightly larger than, yet in good agreement with, the 0.335 nm of AB-stacked graphene.

**Figure S4|** XRD pattern of rGO reduced at 1400 °C (Stage (III)) in (a) argon atmosphere (ethanol partial pressure: 0 Pa) and (b) ethanol atmosphere (ethanol partial pressure: 1.33 Pa). The asterisks indicate peaks originating from the sample holder.

**Table S1|** Interlayer spacing d and FWHM of the 002 diffraction peaks of rGO thin films.

| **Ethanol partial**  **pressure (Pa)** | **d_002_ (nm)** | **FWHM_002_ (deg.)** |
| --- | --- | --- |
| 0 | 0.416 | 7.456 |
| 1.33 | 0.418, 0.340 | 7.269, 0.833 |

In the Raman spectra of multilayer graphene, the 2D band is highly sensitive to interlayer interactions, allowing the stacking structure to be inferred from its spectral features. According to Cançado et al., the 2D band of multilayer graphene can be deconvoluted into three components^9^. The ${G'}_{2D}$ peak located at ~2700 cm^−1^ is associated with a stacking structure characterized by weak interlayer coupling along the c-axis, namely turbostratic stacking. In contrast, the two peaks ${G'}_{3DA}$ (~2680 cm^−1^) and ${G'}_{3DB}$ (~2720 cm^−1^) correspond to a graphite-like structure, i.e., AB-stacking. Equation (S3) provides the expression used to calculate the turbostratic stacking ratio $R'$^9^.

$$\begin{aligned} R^{'} (\%)=1-\frac{I\left( {G^{'}}_{3DB} \right)}{I\left( {G^{'}}_{3DB} \right)-I({G'}_{2D})} \#\left( S3 \right) \end{aligned}$$

Here, $I({G'}_{2D})$, $I\left( {G^{'}}_{3DA} \right)$ and $I\left( {G^{'}}_{3DB} \right)$ denote the intensities of the ${G'}_{2D}$, ${G'}_{3DA}$ and ${G'}_{3DB}$ peaks, respectively. Furthermore, the intensity ratio of the ${G'}_{3DA}$ and ${G'}_{3DB}$ peaks is constant, being ${I\left( {G^{'}}_{3DB} \right)}/{I\left( {G^{'}}_{3DA} \right)\approx2}$. As shown in Fig. 1(a), a pronounced increase in the intensity of the 2D peak is observed at Stage (III). The 2D peak appears clearly as the crystallization of rGO progresses^10-13^. Figure S5(a) shows the fitted 2D peak for rGO reduced at 1400 °C (Stage (III)) in an ethanol atmosphere (ethanol partial pressure: 1.33 Pa). The 2D peak profile exhibits a symmetrical shape, and the experimental spectrum is well reproduced by peak fitting with three Lorentzian functions. Figure S5(b) shows the $R'$ plotted as a function of ethanol partial pressure. $R'$ increases with ethanol partial pressure up to 0.67 Pa and then saturates at approximately 63.4 %. These results indicate that the emergence of the 2D peak is closely linked to the formation of graphene islands. Moreover, the $R'$ value of approximately 63.4 % indicates weak interlayer coupling imposed by the rGO template during the epitaxial growth of graphene islands. Because of this weak interlayer coupling, graphene islands formed on the rGO surface can attain higher crystallinity than the underlying template.

**Figure S5|** (a) Peak fitting of the 2D peak in the Raman spectra of rGO. (b) turbostratic stacking ratio $R'$ as a function of ethanol partial pressure.

**Homoepitaxial growth of graphene**

Figure S6 shows the results of homoepitaxial growth of graphene islands using Cu-CVD–grown graphene as a template. AFM observations reveal the formation of graphene islands with edge structures reflecting the characteristic hexagonal lattice of graphene. This behavior is similar to the graphene growth mechanisms observed in Figs. 2(b, c, e, f), 3(c, f), and 4(a)–(c). These results provide direct crystallographic evidence that the graphene islands are formed via homoepitaxial growth.

**Figure S6|** AFM height image of homoepitaxially grown graphene islands using Cu-CVD–grown graphene as a template.

**Electrical transport properties**

The effective thickness of the rGO thin films varies due to the crystal growth of graphene islands. An increase in the number of layers leads to a decrease in sheet resistance and a corresponding increase in carrier density. Figure S7 shows the carrier density as a function of the number of layers for the rGO thin films measured by Hall effect measurements. While the sheet resistance decreases significantly with increasing number of layers (Fig. 5(c)), the increase in carrier density is relatively modest. This result is attributed to the lower concentration of defect- and impurity-induced carriers in the epitaxially grown layers compared to the rGO template. This interpretation is consistent with the higher crystallinity of the epitaxially grown graphene islands relative to the rGO template.

**Figure S7|** Carrier density as a function of the number of layers in rGO thin films. The carrier density was determined by Hall effect measurements.

**Supplementary references**

1	Vollebregt, S., Ishihara, R., Tichelaar, F. D., Hou, Y. & Beenakker, C. I. M. Influence of the growth temperature on the first and second-order Raman band ratios and widths of carbon nanotubes and fibers. *Carbon* **50**, 3542-3554 (2012). https://doi.org/10.1016/j.carbon.2012.03.026

2	Claramunt, S. *et al.* The importance of interbands on the interpretation of the Raman spectrum of graphene oxide. *J. Phys. Chem. C* **119**, 10123-10129 (2015). <https://doi.org/10.1021/acs.jpcc.5b01590>

3	Lee, A. Y. *et al.* Raman study of D* band in graphene oxide and its correlation with reduction. *Appl. Surf. Sci.* **536**, 147990 (2021).
<https://doi.org/10.1016/j.apsusc.2020.147990>

4	Chen, X., Wang, X. & Fang, D. A review on C1s XPS-spectra for some kinds of carbon materials. *Fuller. Nanotub. Carbon Nanostruct.* **28**, 1048-1058 (2020). <https://doi.org/10.1080/1536383X.2020.1794851>

5	Rozada, R., Paredes, J. I., Villar-Rodil, S., Martínez-Alonso, A. & Tascón, J. M. D. Towards full repair of defects in reduced graphene oxide films by two-step graphitization. *Nano Res.* **6**, 216-233 (2013). <https://doi.org/10.1007/s12274-013-0298-6>

6	Song, L. *et al.* Effect of high-temperature thermal treatment on the structure and adsorption properties of reduced graphene oxide. *Carbon* **52**, 608-612 (2013). <https://doi.org/10.1016/j.carbon.2012.09.060>

7	Gupta, B *et al.* Role of oxygen functional groups in reduced graphene oxide for lubrication. *Sci. Rep.* **7**, 45030 (2017). <https://doi.org/10.1038/srep45030>

8	Kumar, N. & Srivastava, V. C. Simple synthesis of large graphene oxide sheets via electrochemical method coupled with oxidation process. *ACS Omega* **3**, 10233–10242 (2018). <https://doi.org/10.1021/acsomega.8b01283>

9	Cancado, L. G. *et al.* Measuring the degree of stacking order in graphite by Raman spectroscopy. *Carbon* **46**, 272-275 (2008).
<https://doi.org/10.1016/j.carbon.2007.11.015>

10	Ishida, T., Miyata, Y., Shinoda, Y. & Kobayashi, Y. Anomalous restoration of graphitic layers from graphene oxide in ethanol environment at ultrahigh temperature using solar furnace. *Appl. Phys. Express* **9**, 025103 (2016). <https://doi.org/10.7567/Apex.9.025103>

11	De Silva, K. K. H., Huang, H. H., Suzuki, S., Badam, R. & Yoshimura, M. Ethanol-assisted restoration of graphitic structure with simultaneous thermal reduction of graphene oxide. *Jpn. J. Appl. Phys.* **57**, 08NB03 (2018). <https://doi.org/10.7567/Jjap.57.08nb03>

12	Su, C. Y. *et al.* Highly efficient restoration of graphitic structure in graphene oxide using alcohol vapors. *Acs Nano* **4**, 5285-5292 (2010).
<https://doi.org/10.1021/nn101691m>

13	Negishi, R., Nakagiri, T., Akabori, M. & Kobayashi, Y. Promotion of the structural repair of graphene oxide thin films by thermal annealing in water-ethanol vapor. *Thin Solid Films* **775**, 139841 (2023). <https://doi.org/10.1016/j.tsf.2023.139841>
